# TEP versus Lichtenstein: Which technique is better for the repair of primary unilateral inguinal hernias in men?

**DOI:** 10.1007/s00464-015-4603-1

**Published:** 2015-10-21

**Authors:** F. Köckerling, B. Stechemesser, M. Hukauf, A. Kuthe, C. Schug-Pass

**Affiliations:** Department of Surgery and Center for Minimally Invasive Surgery, Academic Teaching Hospital of Charité Medical School, Vivantes Hospital, Neue Bergstrasse 6, 13585 Berlin, Germany; Hernia Center Cologne, PAN – Hospital, Zeppelinstrasse 1, 50667 Cologne, Germany; StatConsult GmbH, Halberstädter Strasse 40 a, 39112 Magdeburg, Germany; Department of General and Visceral Surgery, German Red Cross Hospital, Lützerodestrasse 1, 30161 Hannover, Germany

**Keywords:** TEP, Lichtenstein, Recurrence rate, Pain, Postoperative complications

## Abstract

**Introduction:**

In the update of the guidelines of the European Hernia Society, open Lichtenstein and endoscopic techniques continue to be recommended as the surgical technique of choice for repair of unilateral primary inguinal hernias in men despite the fact that a meta-analysis had identified a higher recurrence rate for TEP compared with Lichtenstein operation. The Guidelines Group had taken that decision because one surgeon in one of the randomized controlled trials included in the meta-analysis had had a very high recurrence rate. Therefore, this study based on registry data now compares the outcome of TEP versus Lichtenstein repair.

**Patients and Methods:**

The analysis of the Herniamed Registry compares the prospective data collected for male patients undergoing primary unilateral inguinal hernia repair using either TEP or open Lichtenstein repair. Inclusion criteria were minimum age of 16 years, male patient, primary unilateral inguinal hernia, elective operation, and availability of data on 1-year follow-up. In total, 17,388 patients were enrolled between September 1, 2009, and August 31, 2013. Of these patients, 10,555 (60.70 %) had a Lichtenstein repair and 6833 (39.30 %) a TEP repair.

**Results:**

On multivariable analysis, the surgical technique was not found to have had any significant effect on the recurrence rate (*p* = 0.146) or on the chronic pain rate (*p* = 0.560). Nor did the complication-related reoperation rates differ significantly between the two techniques (*p* = 0.084). But TEP was found to have benefits as regards the postoperative complication rate (*p* < 0.001), pain at rest rate (*p* = 0.011), and pain on exertion rate (*p* < 0.001).

**Summary:**

In the present registry study, no significant difference was identified in the recurrence rates between the TEP and Lichtenstein technique. TEP was found to have benefits compared with Lichtenstein repair as regards the postoperative complication rates, pain at rest, and pain on exertion.

On the basis of five meta-analyses [1–5], in 2009, the European Hernia Society (EHS) issued in its guidelines recommendations for the treatment of primary unilateral inguinal hernias in men [6]. As Grade A recommendation, it recommended the open Lichtenstein and endoscopic inguinal hernia techniques as the best evidence-based options for the repair of a primary unilateral hernia providing the surgeon was sufficiently experienced in the specific procedure [6].

This was followed in 2012 by a further meta-analysis of the treatment of primary unilateral inguinal hernias in men, which was based on 27 RCTs with a total of 7161 patients [7]. That meta-analysis concluded that TEP was associated with increased risk of recurrence compared with open inguinal hernia repair, but TAPP was not [7]. TAPP was associated with increased risk of perioperative complications compared with open inguinal hernia repair [7]. Endoscopic inguinal hernia repair had a reduced risk of chronic pain and numbness compared with open inguinal hernia repair [7]. Hence, the findings of that meta-analysis call into question the recommendation issued by the EHS in its guidelines published in 2009.

In 2014, an update of the guidelines on the treatment of inguinal hernia in adult patients [8] was published. That update of the guidelines pointed out that due to a Swedish study [9] in which one single surgeon was responsible for 33 % of the TEP recurrences, the difference in recurrence was now significant (*p* = 0.03) in favor of the Lichtenstein technique [8]. Therefore, the Guidelines Group performed the meta-analysis excluding the data from this surgeon in both groups [8]. In that case, the difference in the long-term recurrence rate between Lichtenstein and endoscopic surgery was not significant (*p* = 0.12) [8]. The results for severe chronic pain remained unchanged after inclusion of the Swedish data and did not differ (*p* = 0.34) between the groups [8]. Following in-depth debate in the Guidelines Group, the EHS then decided not to amend the recommendation from 2009 but stressed again the long-learning curve associated with endoscopic repair, especially TEP [8].

It is therefore very important to obtain further results based on comparative studies of Lichtenstein versus TEP repair, in order to be able to confirm or question, on the basis of more data, the findings identified in the meta-analysis by O’Reilly [7].

This paper now analyzes the outcome of TEP versus Lichtenstein repair on the basis of a non-selective patient group from the Herniamed Hernia Registry. Only male patients with primary unilateral inguinal hernia are compared on the basis of the perioperative outcomes and the 1-year follow-up results.

## Patients and methods

The Herniamed quality assurance study is a multicenter, internet-based hernia registry [10] into which 425 participating hospitals and surgeons engaged in private practice (Herniamed Study Group) in Germany, Austria, and Switzerland (status: August 31, 2013) had entered data prospectively on their patients who had undergone hernia surgery. All postoperative complications occurring up to 30 days after surgery are recorded. On 1-year follow-up, postoperative complications are once again reviewed when the general practitioner and patients complete a questionnaire. On 1-year follow-up, general practitioners and patients are also asked about any recurrences, pain at rest, pain on exertion, and chronic pain requiring treatment. This present analysis compares the prospective data collected for all male patients who had undergone primary unilateral inguinal hernia repair using either total extraperitoneal patch plasty (TEP) or open Lichtenstein repair.

Inclusion criteria were minimum age of 16 years, male patient, primary unilateral inguinal hernia, elective operation, lateral or medial EHS classification, and availability of data on 1-year follow-up. In total, 17,388 patients were enrolled between September 1, 2009, and August 31, 2013. Of these patients, 10,555 (60.70 %) had a Lichtenstein repair and 6833 (39.30 %) a TEP repair.

The demographic and surgery-related parameters included age (years), ASA classification (I–IV) as well as the proportion of medial, lateral, and combined EHS classifications and the hernia defect size based on EHS classification (hernia type: medial, lateral, combined. Defect size: Grade I = <1.5 cm, Grade II: 1.5–3 cm, Grade III: >3 cm) [11] and risk factors (nicotine, COPD, diabetes, cortisone, immunosuppression, etc.). Risk factors were dichotomized, i.e., “yes” if at least one risk factor is positive and “no” otherwise. The dependent variables were intra- and postoperative complication rates, reoperation rates, recurrence rates, and rates of pain at rest, pain on exertion, and chronic pain requiring treatment.

All analyses were performed with the software SAS 9.2 (SAS Institute Inc. Cary, NY, USA) and intentionally calculated to a full significance level of 5 %, i.e., they were not corrected in respect of multiple tests, and each *p* value ≤ 0.05 represents a significant result. To discern differences between the groups in unadjusted analyses, Fisher’s exact test was used for categorical outcome variables, and the robust *t* test (Satterthwaite) for continuous variables.

To rule out any confounding of data caused by different patient characteristics, the results of unadjusted analyses were verified via multivariable analyses in which, in addition to operation technique, other influence parameters were simultaneously reviewed, to exclude the factor, that patients with higher age, higher ASA score, greater defect size, and risk factors were more likely to undergo Lichtenstein repair.

To access influence factors in multivariable analyses, the binary logistic regression model for dichotomous outcome variables was used. Estimates for odds ratio (OR) and the corresponding 95 % confidence interval based on the Wald test were given. For influence variables with more than two categories, one of the latter forms was used in each case as reference category. For age (years), the 10-year OR estimate and, for BMI (kg/m^2^), the five-point OR estimate were given. Results are presented in tabular form, sorted by descending impact.

## Results

### Unadjusted analyses

The patients operated on with the Lichtenstein technique were on average 8 years older than those with TEP repair (*p* < 0.001) (Table 1). Furthermore, Lichtenstein repair was significantly more associated with higher ASA scores (ASA III/IV: 24.93 vs 12.34 %), larger hernia defect sizes (EHS classification III: 30.48 vs 18.15 %) as well as medial EHS classification (31.63 vs 18.29 %; Table 2). Hence, more lateral EHS classifications were identified for TEP repair (67.91 vs 50.14 %; Table 2).

**Table 1:** Mean age and BMI with standard deviation

		Operation		
		Lichtenstein	TEP	*p*
Age (years)	Mean ± STD	63.2 ± 15.4	55.3 ± 15.6	<0.001
BMI (kg/m^2^)	Mean ± STD	25.8 ± 3.4	25.7 ± 3.3	0.201

**Table 2:** Distribution of ASA scores, defect sizes, EHS classifications, and risk factors

	TEP		Lichtenstein		*p*
	*n*	%	*n*	%	
ASA score					
1	2294	33.57	2980	28.23	<0.001
II	3696	54.09	4944	46.84	
III/IV	843	12.34	2631	24.93	
Defect size					
I	1182	17.30	1344	12.73	<0.001
II	4411	64.55	5994	56.79	
III	1240	18.15	3217	30.48	
EHS classification					
Medial	1250	18.29	3339	31.63	<0.001
Lateral	4640	67.91	5292	50.14	
Combined	943	13.80	1924	18.23	
Risk factors					
Total					
Yes	1765	25.83	4136	39.19	<0.001
No	5068	74.17	6419	60.81	
COPD					
Yes	303	4.43	896	8.49	<0.001
No	6530	95.57	9659	91.51	
Diabetes					
Yes	299	4.38	893	8.46	<0.001
No	6534	96.52	9662	91.54	
Aortic aneurism					
Yes	21	0.31	125	1.18	<0.001
No	6812	99.69	10430	98.82	
Immunosuppression					
Yes	29	0.42	137	1.30	<0.001
No	6804	99.58	10,418	98.70	
Corticoids					
Yes	68	1.00	148	1.40	0.020
No	6765	99.00	10,407	98.60	
Smoking					
Yes	789	11.55	1250	11.84	0.563
No	6044	88.45	9305	88.16	
Coagulopathy					
Yes	80	1.17	235	2.23	<0.001
No	6753	98.83	10,320	97.77	
Antiplatelet medication					
Yes	440	6.44	1410	13.36	<0.001
No	6393	93.56	9145	86.64	
Anticoagulation therapy					
Yes	96	1.40	503	4.77	<0.001
No	6737	98.60	10,052	95.23	

A global view of the risk factors, i.e., the presence of at least one risk factor, shows an equally significant difference between TEP and Lichtenstein repair (*p* < 0.001). Up to 25.83 % of patients with TEP repair had at least one risk factor, while that figure amounted to 39.19 % for those with Lichtenstein repair (Table 2). Equally, for most individual risk factors, the corresponding rate was significantly higher among patients with Lichtenstein repair (Table 2). That applies in particular for the highly significant greater proportion of patients with coagulopathy and on antiplatelet therapy and coumarin-derivative therapy (Table 2).

Unadjusted analysis of the two surgical techniques revealed that there was no difference in the overall rate of intraoperative complications (Table 3). Conversely, major differences were noted in the postoperative complications at the expense of the Lichtenstein technique (Table 3).

**Table 3:** Unadjusted analysis of intra- and postoperative complications, reoperations, and pain and recurrence rates on 1-year follow-up

	TEP		Lichtenstein		*p*
	*n*	%	*n*	%	
*Intraoperative complications*					
Total					
Yes	80	1.17	133	1.26	0.622
No	6753	98.83	10,422	98.74	
Bleeding					
Yes	52	0.76	43	0.41	0.003
No	6781	99.24	10,512	99.59	
Injuries					
Total					
Yes	43	0.63	103	0.98	0.017
No	6790	99.37	10,452	99.02	
Vascular					
Yes	19	0.28	15	0.14	0.054
No	6814	99.72	10,540	99.86	
Bowell					
Yes	4	0.06	5	0.05	0.745
No	6829	99.94	10,550	99.95	
Bladder					
Yes	3	0.04	3	0.03	0.685
No	6830	99.96	10,552	99.97	
Nerve					
Yes	0	0.00	65	0.62	<0.001
No	6833	100.0	10,490	99.38	
*Postoperative complications*					
Total					
Yes	115	1.68	447	4.23	<0.001
No	6718	98.32	10,108	95.77	
Bleeding					
Yes	79	1.16	260	2.46	<0.001
No	6754	98.84	10,295	97.54	
Seroma					
Yes	35	0.51	156	1.48	<0.001
No	6798	99.49	10,399	98.52	
Infection					
Yes	4	0.06	27	0.26	0.003
No	6829	99.94	10,528	99.74	
Bowell injury/anastomotic leakage					
Yes	0	0.00	2	0.02	0.523
No	6833	100.0	10,553	99.98	
Wound healing disorders					
Yes	5	0.07	37	0.35	<0.001
No	6828	99.93	10,518	99.35	
Ileus					
Yes	0	0.00	2	0.02	0.523
No	6833	100.0	10,553	99.98	
*Reoperations*					
Yes	49	0.72	138	1.31	<0.001
No	6784	99.28	10,417	98.69	
*Recurrence on follow-up*					
Yes	64	0.94	88	0.83	0.505
No	6769	99.06	10,467	99.17	
*Pain at rest on follow-up*					
Yes	276	4.04	487	4.61	0.075
No	6557	95.96	10,038	95.39	
*Pain on exertion on follow-up*					
Yes	540	7.90	974	9.23	0.002
No	6293	92.10	9581	90.77	
*Pain requiring treatment*					
Yes	160	2.34	242	2.29	0.836
No	6673	97.36	10,313	97.71	

For example, there was a highly significant difference in the overall postoperative complication rate, which was 4.23 % for the Lichtenstein operation versus 1.68 % for TEP repair (*p* < 0.001). That difference was attributable to a higher secondary bleeding rate (2.46 vs 1.16 %; *p* < 0.001), higher seroma rate (1.48 vs 0.51 %; *p* < 0.001), higher impaired wound healing rate (0.35 vs 0.07 %; *p* < 0.001), and higher mesh infection rate (0.26 vs 0.06 %; *p* = 0.003) at the expense of the Lichtenstein technique (Table 3).

Because of the higher postoperative complication rate, more reoperations were also performed after the Lichtenstein operation (1.31 vs 0.72 %; *p* < 0.001) (Table 3).

On 1-year follow-up, no differences were observed in the recurrence rate (Table 3), pain at rest rate, or in the rate of chronic pain requiring treatment. Only for the pain on exertion rate was a higher score identified for the Lichtenstein operation (9.23 vs 7.90 %; *p* = 0.002).

### Multivariable analysis

#### Intraoperative complications

Model matching for analysis of the intraoperative complication rate, which reflects the suitability of the influence parameters to explain the target variable scores, was not significant (*p* = 0.910). As such, there was no evidence of the individual variables having influenced the intraoperative complication rate.

#### Postoperative complications

Multivariable analysis confirmed (model matching: *p* < 0.001) that the risk of onset of a postoperative complication was primarily influenced by the surgical technique (*p* < 0.001). The overall risk of a postoperative complication was significantly increased by the use of the Lichtenstein technique (OR 2.152 [1.734; 2.672]) (Table 4). With a prevalence of 3.2 %, that would correspond to 44 postoperative complications for every 1000 patients with Lichtenstein repair versus 21 complications for patients with TEP repair.

**Table 4:** Multivariable analysis of postoperative complications

Parameter	*p* value	Category	OR estimate	95 % CI	
Operation	<0.001	Lichtenstein versus TEP	2.152	1.734	2.672
Age [10-year OR]	<0.001		1.148	1.069	1.232
ASA score	<0.001	II versus I	0.980	0.768	1.252
		III/IV versus I	1.483	1.105	1.990
Risk factors	0.007	Yes versus no	1.295	1.075	1.561
BMI [5-point OR]	0.153		0.909	0.797	1.036
EHS classification	0.354	Lateral versus combined	1.184	0.930	1.507
		Medial versus combined	1.088	0.834	1.419
Defect size	0.427	II versus	0.844	0.654	1.089
		III versus	0.873	0.656	1.163

Likewise, higher age (10-year OR 1.148 [1.069; 1.232]) and higher ASA score (III/IV vs I: OR 1.483 [1.105; 1.990]) were highly significantly associated with onset of a postoperative complication (in each case *p* < 0.001). Finally, the presence of a risk factor (OR 1.295 [1.075; 1.561]; *p* = 0.007) also resulted in significantly more postoperative complications.

#### Reoperation rate

Multivariable analysis of the reoperation rate (model matching: *p* < 0.001) identified the ASA score as being the most powerful influence factor (*p* < 0.001; Table 5). Patients with a higher ASA score (III/IV vs I: OR 2.174 [1.297; 3.642]) were at increased risk of reoperation. Equally, higher age (10-year OR 1.219 [1.073; 1.386]; *p* = 0.002) and the presence of a risk factor (OR 1.409 [1.021; 1.945]; *p* = 0.037) significantly increased the reoperation rate. However, there was no evidence of the surgical technique having influenced the reoperation rate.

**Table 5:** Multivariable analysis of reoperation

Parameter	*p* value	Category	OR estimate	95 % CI	
ASA score	<0.001	II versus I	0.890	0.562	1.408
		III/IV versus I	2.174	1.297	3.642
Age [10-year OR]	0.002		1.219	1.073	1.386
Risk factors	0.037	Yes versus no	1.409	1.021	1.945
EHS classification	0.055	Lateral versus combined	0.999	0.685	1.457
		Medial versus combined	0.633	0.399	1.003
Operation	0.084	Lichtenstein versus TEP	1.356	0.960	1.913
BMI [5-point OR]	0.089		0.819	0.651	1.031
Defect size	0.522	II versus I	0.778	0.505	1.198
		III versus I	0.812	0.500	1.319

#### Recurrence

For the recurrence rate (model matching: *p* < 0.001), the BMI was shown be to the most powerful influence factor (*p* < 0.001; Table 6). A five-point higher BMI led to an increase in the recurrence rate (five-point OR 1.439 [1.183; 1.750]). Likewise, the EHS classification had a significant impact on the recurrence rate (*p* = 0.013). Medial EHS classification resulted in a higher recurrence rate (OR 1.417 [0.881; 2.281]). However, on 1-year follow-up, there was no evidence of the surgical technique having impacted the recurrence rate.

**Table 6:** Multivariable analysis of recurrence

Parameter	*p* value	Category	OR estimate	95 % CI	
BMI [5-point OR]	<0.001		1.439	1.183	1.750
EHS classification	0.013	Lateral versus combined	0.821	0.515	1.306
		Medial versus combined	1.417	0.881	2.281
Operation	0.146	Lichtenstein versus TEP	0.775	0.549	1.093
ASA score	0.240	II versus I	1.293	0.837	1.997
		III/IV versus I	1.637	0.924	2.898
Defect size	0.349	II versus I	0.744	0.468	1.184
		III versus I	0.905	0.539	1.519
Age [10-year OR]	0.749		0.980	0.864	1.111
Risk factors	0.981	Yes versus no	1.004	0.700	1.442

#### Pain at rest

For pain at rest, on 1-year follow-up (model matching: *p* < 0.001), the hernia defect size proved to be the most powerful influence factor (*p* < 0.001; Table 7). A larger hernia defect size reduced the risk of pain at rest (II vs I: OR 0.694 [0.571; 0.842]; III vs I: OR 0.631 [0.498; 0.799]). Equally, the BMI had a highly significant impact on pain at rest. A five-point higher BMI increased the risk of onset of pain at rest (five-point OR 1.206 [1.088; 1.336]). Furthermore, the risk of pain at rest rose on using the Lichtenstein technique (OR 1.231 [1.049; 1.444]; *p* = 0.011). With an overall prevalence of 4.4 %, that would correspond to 48 patients with pain at rest for every 1000 Lichtenstein operation versus 40 out of every 1000 patients with TEP repair. But higher age reduced the risk of pain at rest (OR 0.945 [0.894; 0.998]; *p* = 0.043).

**Table 7:** Multivariable analysis of pain at rest

Parameter	*p* value	Category	OR estimate	95 % CI	
Defect size	<0.001	II versus I	0.694	0.571	0.842
		III versus I	0.631	0.498	0.799
BMI [5-point OR]	<0.001		1.206	1.088	1.336
Operation	0.011	Lichtenstein versus TEP	1.231	1.049	1.444
Age [10-year OR]	0.043		0.945	0.894	0.998
EHS classification	0.680	Lateral versus combined	1.047	0.847	1.295
		Medial versus combined	1.106	0.876	1.396
ASA score	0.876	II versus I	1.034	0.860	1.244
		III/IV versus I	0.989	0.759	1.288
Risk factor	0.982	Yes versus no	0.998	0.844	1.180

#### Pain on exertion

Pain on exertion on 1-year follow-up (model matching: *p* < 0.001) was highly significantly influenced by the surgical technique (*p* < 0.001) (Table 8). The use of the Lichtenstein technique (OR 1.420 [1.264; 1.596]) was conducive to onset of pain on exertion. With an overall prevalence of 8.7 %, that would amount to onset of pain on exertion in around 102 out of every 1000 patients with Lichtenstein repair versus 74 out of every 1000 patients with TEP repair.

**Table 8:** Multivariable analysis of pain on exertion

Parameter	*p* value	Category	OR estimate	95 % CI	
Operation	<0.001	Lichtenstein versus TEP	1.420	1.264	1.596
Age [10-year OR]	<0.001		0.831	0.799	0.864
Defect size	<0.001	II versus I	0.763	0.662	0.879
		III versus I	0.598	0.501	0.714
BMI [5-point OR]	<0.001		1.162	1.077	1.254
EHS classification	0.054	Lateral versus combined	1.090	0.930	1.279
		Medical versus combined	1.222	1.028	1.452
ASA score	0.112	II versus I	1.066	0.935	1.216
		III/IV versus I	0.902	0.739	1.101
Risk factors	0.343	yes versus no	1.061	0.939	1.199

Likewise, higher age, hernia defect size, and BMI had a highly significant effect on pain on exertion (in each case *p* < 0.001). Higher age (10-year OR 0.831 [0.799; 0.864]) and larger hernia defect size (II vs I: OR 0.763 [0.662; 0.879]; III vs I: OR 0.598 [0.501; 0.714]) reduced onset of pain on exertion. Conversely, the risk of pain rose in line with a five-point higher BMI (five-point OR 1.162 [1.077; 1.254]).

#### Chronic pain requiring treatment

For chronic pain requiring treatment (model matching: *p* < 0.001), BMI was identified as being the most powerful influence factor (*p* < 0.001; Table 9). A five-point higher BMI increased the rate of chronic pain requiring treatment (five-point OR 1.276 [1.118; 1.455]). Likewise, the ASA score had a significant influence (*p* = 0.017) on increased risk of chronic pain requiring treatment (II vs I: OR 1.397 [1.078; 1.810]; III/IV vs I: OR 1.620 [1.130; 2.325]).

**Table 9:** Multivariable analysis of pain requiring treatment

Parameter	*p* value	Category	OR estimate	95 % CI	
BMI [5-point OR]	<0.001		1.276	1.118	1.455
Age [10-year OR]	<0.001		0.850	0.790	0.916
Defect size	<0.001	II versus I	0.598	0.464	0.770
		III versus I	0.515	0.376	0.707
ASA score	0.017	II versus I	1.397	1.078	1.810
		III/IV versus I	1.620	1.130	2.325
Risk factors	0.185	Yes versus no	1.162	0.930	1.452
EHS classification	0.327	Lateral versus combined	0.957	0.716	1.278
		Medial versus combined	1.143	0.836	1.564
Operation	0.560	Lichtenstein versus TEP	1.066	0.860	1.321

Higher age (10-year OR 0.850 [0.790; 0.916]; *p* < 0.001) and larger hernia defect size (II vs I: OR 0.598 [0.464; 0.770]; III vs I: OR 0.515 [0.376; 0.707]; *p* < 0.001) reduced the risk of chronic pain requiring treatment. There was no evidence of the surgical technique having impacted the rate of chronic pain requiring treatment (*p* = 0.560).

## Discussion

This paper reports on analysis of a non-selective patient group from the Herniamed Hernia Registry aimed at identifying whether there are any significant differences in the perioperative outcome and 1-year follow-up between the TEP and Lichtenstein techniques when used to repair primary unilateral inguinal hernias in men. The surgical technique was not found to have any significant influence on the intraoperative complication rate, complication-related reoperation rate, chronic pain rate requiring treatment, or recurrence rate. Hence, on comparing 10,555 primary unilateral inguinal hernias in men with Lichtenstein repair versus 6833 with TEP repair, multivariable analysis, which can rule out other influence factors like higher patient age, higher ASA score, greater defect size, and risk factors, did not find any evidence that the surgical technique had any influence on the recurrence rate. Instead, the influence factors identified were higher BMI and medial EHS classification.

Nor was the surgical technique found to have any influence on onset of chronic pain requiring treatment; rather, this was negatively influenced by a high BMI and ASA score. Chronic pain requiring treatment occurred less often in patients with higher age and larger defects. The complication-related reoperation rate was found to be associated with a high ASA score, higher patient age, and the presence of risk factors.

Matters were different for the postoperative complications, pain at rest, and pain on exertion. Multivariable analysis revealed that these rates were significantly affected by the surgical method, in addition to other influence factors characterizing a “bad” hernia, with the Lichtenstein technique having a negative effect. The postoperative complications were also adversely affected by high age, higher ASA score, and the presence of risk factors. However, since no significant difference was found between the TEP and Lichtenstein technique as regards the complication-related reoperation rate, the significant difference identified here between the TEP and Lichtenstein technique related only to the conservatively treated postoperative complications.

Pain at rest occurred significantly more often after repair of small defects, in the presence of a higher BMI and following Lichtenstein operation. That was also true for pain on exertion. Besides, pain on exertion was significantly less common in older patients.

If one compares these findings with those of the meta-analysis by O’Reilly et al. [7], other differences are identified in addition to the recurrence rate. The meta-analysis did not find any significant difference in the perioperative surgical risk or chronic pain rate between the TEP and Lichtenstein operation. That may be partly due to the use of different definitions in the various studies included in the meta-analysis and the registry analysis presented here. In this present analysis, no significant difference was detected either between the TEP and Lichtenstein operation with regard to the postoperative complications necessitating reoperation or the chronic pain rates requiring treatment. Significant differences, in favor of TEP operation, were identified only for the conservatively treated postoperative complications and the occasional pain at rest and pain on exertion not requiring treatment.

In summary, it can be stated that the endoscopic TEP and the open Lichtenstein operation had comparable recurrence rates, reoperation rates for postoperative complications, and chronic pain requiring treatment. Benefits were identified for TEP in terms of postoperative complications with the need for conservative treatment and pain at rest and pain on exertion. The findings of this present registry study thus confirm the validity of the decision taken by the Guidelines Group of the European Hernia Society to continue to recommend open Lichtenstein and endoscopic techniques for repair of unilateral primary inguinal hernias in men.

## Appendix: Herniamed Study Group

### Scientific board

**Köckerling**, Ferdinand (Chairman) (Berlin); **Berger**, Dieter (Baden–Baden); **Bittner**, Reinhard (Rottenburg); **Bruns**, Christiane (Magdeburg); **Dalicho**, Stephan (Magdeburg); **Fortelny**, René (Wien); **Jacob**, Dietmar (Berlin); **Koch**, Andreas (Cottbus); **Kraft,** Barbara (Stuttgart); **Kuthe**, Andreas (Hannover); **Lippert**, Hans (Magdeburg): **Lorenz**, Ralph (Berlin); **Mayer**, Franz (Salzburg); **Moesta**, Kurt Thomas (Hannover); **Niebuhr**, Henning (Hamburg); **Peiper**, Christian (Hamm); **Pross**, Matthias (Berlin); **Reinpold**, Wolfgang (Hamburg); **Simon**, Thomas (Sinsheim); **Stechemesser**, Bernd (Köln); **Unger**, Solveig (Chemnitz).

### Participants

**Ahmetov**, Azat (Saint-Petersburg); **Alapatt,** Terence Francis (Frankfurt/Main); **Anders,** Stefan (Berlin); **Anderson**, Jürina (Würzburg); **Arndt**, Anatoli (Elmshorn); **Asperger,** Walter (Halle); **Avram**, Iulian (Saarbrücken); **Barkus**; Jörg (Velbert); **Becker**, Matthias (Freital); **Behrend**, Matthias (Deggendorf); **Beuleke,** Andrea (Burgwedel); **Berger,** Dieter (Baden–Baden); **Bittner,** Reinhard (Rottenburg); **Blaha,** Pavel (Zwiesel); **Blumberg,** Claus (Lübeck); **Böckmann,** Ulrich (Papenburg); **Böhle**, Arnd Steffen (Bremen); **Böttger,** Thomas Carsten (Fürth); **Bolle**, Ludger (Berlin); **Borchert,** Erika (Grevenbroich); **Born**, Henry (Leipzig); **Brabender,** Jan (Köln); **Brauckmann,** Markus (Rüdesheim am Rhein); **Breitenbuch von**, Philipp (Radebeul); **Brüggemann**, Armin (Kassel); **Brütting**, Alfred (Erlangen); **Budzier**, Eckhard (Meldorf); **Burghardt**, Jens (Rüdersdorf); **Carus**, Thomas (Bremen); **Cejnar**, Stephan-Alexander (München); **Chirikov**, Ruslan (Dorsten); **Comman,** Andreas (Bogen); **Crescent**i, Fabio (Verden/Aller); **Dapunt**, Emanuela (Bruneck); **Decker**, Georg (Berlin); **Demmel**, Michael (Arnsberg); **Descloux,** Alexandre (Baden); **Deusch**, Klaus-Peter (Wiesbaden); **Dick**, Marcus (Neumünster); **Dieterich**, Klaus (Ditzingen); **Dietz**, Harald (Landshut); **Dittmann**, Michael (Northeim); **Dornbusch**, Jan (Herzberg/Elster); **Drummer**, Bernhard (Forchheim); **Eckermann**, Oliver (Luckenwalde); **Eckhoff,** Jörn/Hamburg); **Elger**, Karlheinz (Germersheim); **Engelhardt**, Thomas (Erfurt); **Erichsen,** Axel (Friedrichshafen); **Eucker**, Dietmar (Bruderholz); **Fackeldey**, Volker (Kitzingen); **Farke**, Stefan (Delmenhorst); **Faust**, Hendrik (Emden); **Federmann**, Georg (Seehausen); **Feichter**, Albert (Wien); **Fiedler,** Michael (Eisenberg); **Fischer**, Ines (Wiener Neustadt); **Fortelny**, René H. (Wien); **Franczak**, Andreas (Wien); **Franke**, Claus (Düsseldorf); **Frankenberg von**, Moritz (Salem); **Frehner**, Wolfgang (Ottobeuren); **Friedhoff**, Klaus (Andernach); **Friedrich,** Jürgen (Essen); **Frings**, Wolfram (Bonn); **Fritsche**, Ralf (Darmstadt); **Frommhold,** Klaus (Coesfeld); **Frunder**, Albrecht (Tübingen); **Fuhrer**, Günther (Reutlingen); **Gassler,** Harald (Villach); **Gawad**, Karim A. Frankfurt/Main); **Gerdes**, Martin (Ostercappeln); **Germanov**, German (Halberstadt; **Gilg**, Kai-Uwe (Hartmannsdorf); **Glaubitz**, Martin (Neumünster); **Glutig,** Holger (Meissen); **Gmeiner**, Dietmar (Bad Dürrnberg); **Göring**, Herbert (München); **Grebe**, Werner (Rheda-Wiedenbrück); **Grothe**, Dirk (Melle); **Gürtler**, Thomas (Zürich); **Hache**, Helmer (Löbau); **Hämmerle**, Alexander (Bad Pyrmont); **Haffner**, Eugen (Hamm); **Hain**, Hans-Jürgen (Gross-Umstadt); **Hammans**, Sebastian (Lingen); **Hampe**, Carsten (Garbsen); **Harrer**, Petra (Starnberg); **Heinzmann**, Bernd (Magdeburg); **Heise**, Joachim Wilfried (Stolberg); **Heitland**, Tim (München); **Helbling**, Christian (Rapperswil); **Hempen**, Hans-Günther (Cloppenburg); **Henneking**, Klaus-Wilhelm (Bayreuth); **Hennes**, Norbert (Duisburg); **Hermes**, Wolfgang (Weyhe); **Herrgesell**, Holger (Berlin); **Herzing**, Holger Höchstadt); **Hessler**, Christian (Bingen); **Hildebrand**, Christiaan (Langenfeld); **Höferlin**, Andreas (Mainz); **Hoffmann**, Henry (Basel); **Hoffmann**, Michael (Kassel); **Hofmann**, Eva M. (Frankfurt/Main); **Hopfe**r, Frank (Eggenfelden); **Hornung**, Frederic (Wolfratshausen); **Hügel**, Omar (Hannover); **Hüttemann**, Martin (Oberhausen); **Huhn**, Ulla (Berlin); **Hunkeler**, Rolf (Zürich); **Imdahl**, Andreas (Heidenheim); **Jacob**, Dietmar (Berlin); **Jenert**, Burghard (Lichtenstein); **Jugenheimer**, Michael (Herrenberg); **Junger**, Marc (München); **Kaaden**, Stephan (Neustadt am Rübenberge); **Käs**, Stephan (Weiden); **Kahraman,** Orhan (Hamburg); **Kaiser,** Christian (Westerstede); **Kaiser**, Stefan (Kleinmachnow); **Kapischke**, Matthias (Hamburg); **Karch**, Matthias (Eichstätt); **Kasparek**, Michael S. (München); **Keck**, Heinrich (Wolfenbüttel); **Keller,** Hans W. (Bonn); **Kienzle**, Ulrich (Karlsruhe); **Kipfmüller**, Brigitte (Köthen); **Kirsch**, Ulrike (Oranienburg); **Klammer**, Frank (Ahlen); **Klatt**, Richard (Hagen); **Kleemann**, Nils (Perleberg); **Klein**, Karl-Hermann (Burbach); **Kleist**, Sven (Berlin); **Klobusicky**, Pavol (Bad Kissingen); **Kneifel**, Thomas (Datteln); **Knoop**, Michael (Frankfurt/Oder); **Knotter**, Bianca (Mannheim); **Koch,** Andreas (Cottbus); **Köckerling,** Ferdinand (Berlin); **Köhler**, Gernot (Linz); **König**, Oliver (Buchholz); **Kornblum**, Hans (Tübingen); **Krämer**, Dirk (Bad Zwischenahn); **Kraft**, Barbara (Stuttgart); **Kreissl**, Peter (Ebersberg); **Krones**, Carsten Johannes (Aachen); **Kruse,** Christinan (Aschaffenburg); **Kube**, Rainer (Cottbus); **Kühlberg**, Thomas (Berlin); **Kuhn,** Roger (Gifhorn); **Kusch,** Eduard (Gütersloh); **Kuthe,** Andreas (Hannover); **Ladberg**, Ralf (Bremen); **Ladra**, Jürgen (Düren); **Lahr-Eigen**, Rolf (Potsdam); **Lainka**, Martin (Wattenscheid); **Lammers**, Bernhard J. (Neuss); **Lancee**, Steffen (Alsfeld); **Lange**, Claas (Berlin); **Laps**, Rainer (Ehringshausen); **Larusson**, Hannes Jon (Pinneberg); **Lauschke**, Holger (Duisburg); **Leher,** Markus (Schärding); **Leidl**, Stefan (Waidhofen/Ybbs); **Lenz**, Stefan (Berlin); **Lesch**, Alexander (Kamp-Lintfort); **Liedke**, Marc Olaf (Heide); **Lienert**, Mark (Duisburg); **Limberger**, Andreas (Schrobenhausen); **Limmer**, Stefan (Würzburg); **Locher**, Martin (Kiel); **Loghmanieh**, Siawasch (Viersen); **Lorenz**, Ralph (Berlin); **Mallmann**, Bernhard (Krefeld); **Manger**, Regina (Schwabmünchen); **Maurer**, Stephan (Münster); **Mayer**, Franz (Salzburg); **Mellert**, Joachim (Höxter); **Menzel**, Ingo (Weimar); **Meurer**, Kirsten (Bochum); **Meyer**, Moritz (Ahaus**)**; **Mirow**, Lutz (Kirchberg); **Mittenzwey**, Hans-Joachim (Berlin); **Mörder-Köttgen**, Anja (Freiburg); **Moesta**, Kurt Thomas (Hannover); Moldenhauer, Ingolf (Braunschweig); **Morkramer**, Rolf (Xanten); **Mosa**, Tawfik (Merseburg); **Müller**, Hannes (Schlanders); **Münzberg**, Gregor (Berlin); **Mussack,** Thomas (St. Gallen); **Neumann**, Jürgen (Haan); **Neumeuer**, Kai (Paderborn); **Niebuhr,** Henning (Hamburg); **Nix**, Carsten (Walsrode); **Nölling**, Anke (Burbach); **Nostitz**, Friedrich Zoltán (Mühlhausen); **Obermaier**, Straubing); **Öz-Schmidt**, Meryem (Hanau); **Oldorf**, Peter (Usingen); **Olivieri**, Manuel (Pforzheim); **Pawelzik**, Marek (Hamburg); **Peiper**, Christian (Hamm); **Peitgen**, Klaus (Bottrop); **Pertl**, Alexander (Spittal/Drau); **Philipp**, Mark (Rostock); **Pickart**, Lutz (Bad Langensalza); **Pizzera**, Christian (Graz); **Pöllath**, Martin (Sulzbach-Rosenberg); **Possin**, Ulrich (Laatzen); **Prenzel**, Klaus (Bad Neuenahr-Ahrweiler); **Pröve**, Florian (Goslar); **Pronnet**, Thomas (Fürstenfeldbruck); **Pross**, Matthias (Berlin); **Puff**, Johannes (Dinkelsbühl); **Rabl**, Anton (Passau); **Rapp**, Martin (Neunkirchen); **Reck**, Thomas (Püttlingen); **Reinpold,** Wolfgang (Hamburg); **Reuter**, Christoph (Quakenbrück); **Richter,** Jörg (Winnenden); **Riemann**, Kerstin (Alzenau-Wasserlos); **Rodehorst**, Anette (Otterndorf); **Roehr**, Thomas (Rödental); **Roncossek**, Bremerhaven); **Roth** Hartmut (Nürnberg); **Sardoschau**, Nihad (Saarbrücken); **Sauer**, Gottfried (Rüsselsheim); **Sauer**, Jörg (Arnsberg); **Seekamp**, Axel (Freiburg); **Seelig**, Matthias (Bad Soden); **Seidel**, Hanka (Eschweiler); **Seiler**, Christoph Michael (Warendorf); **Seltmann,** Cornelia (Hachenburg); **Senkal,** Metin (Witten); **Shamiyeh**, Andreas (Linz); **Shang**, Edward (München); **Siemssen**, Björn (Berlin); **Sievers,** Dörte (Hamburg); **Silbernik**, Daniel (Bonn); **Simon**, Thomas (Sinsheim); **Sinn**, Daniel (Olpe); **Sinning**, Frank (Nürnberg); **Smaxwil**, Constatin Aurel (Stuttgart); **Schabel**, Volker (Kirchheim/Teck); **Schadd**, Peter (Euskirchen); **Schassen von**, Christian (Hamburg); **Schattenhofer**, Thomas (Vilshofen); **Scheidbach**, Hubert (Neustadt/Saale); **Schelp**, Lothar (Wuppertal); **Scherf**, Alexander (Pforzheim); **Scheyer**, Mathias (Bludenz); **Schimmelpenning,** Hendrik (Neustadt in Holstein); **Schinkel**, Svenja (Kempten); **Schmid**, Michael (Gera); **Schmid,** Thomas (Innsbruck); **Schmidt**, Rainer (Paderborn); **Schmidt**, Sven-Christian (Berlin); **Schmidt,** Ulf (Mechernich); **Schmitz**, Heiner (Jena); **Schmitz**, Ronald (Altenburg); **Schöche**, Jan (Borna); **Schoenen**, Detlef (Schwandorf); **Schrittwieser**, Rudolf/Bruck an der Mur); **Schroll**, Andreas (München); **Schultz**, Christian (Bremen-Lesum); **Schultz**, Harald (Landstuhl); **Schulze**, Frank P. Mülheim an der Ruhr); **Schumacher**, Franz-Josef (Oberhausen); **Schwab**, Robert (Koblenz); **Schwandner**, Thilo (Lich); **Schwarz**, Jochen Günter (Rottenburg); **Schymatzek**, Ulrich (Radevormwald); **Spangenberger**, Wolfgang (Bergisch-Gladbach); **Sperling**, Peter (Montabaur); **Staade**, Katja (Düsseldorf); **Staib**, Ludger (Esslingen); **Stamm**, Ingrid (Heppenheim); **Stark**, Wolfgang (Roth); **Stechemesser,** Bernd (Köln); **Steinhilper**, Uz (München); **Stengl**, Wolfgang (Nürnberg); **Stern**, Oliver (Hamburg); **Stöltzing**, Oliver (Meißen); **Stolte**, Thomas (Mannheim); **Stopinski,** Jürgen (Schwalmstadt); **Stubbe**, Hendrik (Güstrow/); **Stülzebach**, Carsten (Friedrichroda); **Tepel,** Jürgen (Osnabrück); **Terzić**, Alexander (Wildeshausen); **Teske,** Ulrich (Essen); **Thews**, Andreas (Schönebeck); **Tichomirow,** Alexej (Brühl); **Tillenburg**, Wolfgang (Marktheidenfeld); **Timmermann,** Wolfgang (Hagen); **Tomov**, Tsvetomir (Koblenz; **Train**, Stefan H. (Gronau); **Trauzettel**, Uwe (Plettenberg); **Triechelt**, Uwe (Langenhagen); **Ulcar**, Heimo (Schwarzach im Pongau); **Unger**, Solveig (Chemnitz); **Verweel**, Rainer (Hürth); **Vogel**, Ulrike (Berlin); **Voigt**, Rigo (Altenburg); **Voit**, Gerhard (Fürth); **Volkers**, Hans-Uwe (Norden); **Vossough**, Alexander (Neuss); **Wallasch**, Andreas (Menden); **Wallner**, Axel (Lüdinghausen); **Warscher,** Manfred (Lienz); **Warwas**, Markus (Bonn); **Weber**, Jörg (Köln); **Weihrauch**, Thomas (Ilmenau); **Weiß**, Johannes (Schwetzingen); **Weißenbach**, Peter (Neunkirchen); **Werner**, Uwe (Lübbecke-Rahden); **Wessel,** Ina (Duisburg); **Weyhe**, Dirk (Oldenburg); **Wieber**, Isabell (Köln); **Wiesmann**, Aloys (Rheine); **Wiesner**, Ingo (Halle); **Withöft**, Detlef (Neutraubling); **Woehe**, Fritz (Sanderhausen); **Wolf**, Claudio (Neuwied); **Yaksan**, Arif (Wermeskirchen); **Yildirim**, Selcuk (Berlin); **Zarras**, Konstantinos (Düsseldorf); **Zeller**, Johannes (Waldshut-Tiengen); **Zhorzel**, Sven (Agatharied); **Zuz**, Gerhard (Leipzig);
